# Well-Marked and Severe Chicken Pox Occurring in an Adult Pregnant Woman

**Published:** 1896-05-02

**Authors:** George Mackern

**Affiliations:** late Visiting Physician, British Hospital, Buenos Ayres


### WELL-MARKED AND SEVERE CHICKEN POX OCCURRING IN AN ADULT PREGNANT WOMAN.

By George Mackern, M.D.Lond., late Visiting Physician, British Hospital, Buenos Ayres.

On a Friday I was asked to call and see a woman said to be suffering from measles, the eruption having suddenly appeared the evening before. The patient was a Spanish woman, aged 27, and pregnant seven months with her first child; she had always enjoyed good health, and all through the pregnancy until now had not suffered from sickness. The history of the case showed that on the Monday (five days ago) she had felt her stomach "hot," as she termed it, and took a mild saline aperient. She went about her work as usual, feeling fairly well till the Thursday morning, when she got up, complaining of great pain in her back, nausea, and general malaise. The symptoms increased, so that by the afternoon she was very sick, vomiting and retching every few minutes, and with considerable headache in addition to the lumbar pain.

In the evening she first noticed a red rash round her neck, which gradually passed down to her chest, spreading, during the night, to the abdomen, back, buttocks, and upper parts of thighs. She continued vomiting at intervals during the night, and was very restless, owing to great itching of the skin. Next morning the rash had appeared on the face and upper arms.

I saw her about half-past four p.m., and on first sight it was difficult to think of anything else except modified or discrete small-pox. The face was covered with red papules, which, when the finger was passed lightly over them, gave a distinct "shot-like" sensation to the skin, especially on the forehead. (I regret that I omitted to try Gee's test—stretching the skin—when the shot-like feeling disappears.) The face was flushed, the patient ill and restless, and there was constant vomiting and lumbar pain, with a prodromal history of four days' duration. She said she had been vaccinated when an infant, but I could find no cicatrices on the arms, and this, of course, further complicated the case. On inspecting the chest, however, the nature of the eruption revealed the case. The skin between the breasts and on the mammæ themselves was covered with typical chicken-pox in its various stages. Vesicles as large as small peas, and filled with bright, translucent liquid, stood out from a scarcely inflamed base, looking like glass beads, so thin was the vesicular wall. Other and smaller vesicles had already become turbid, and some were somewhat umbilicated, but there was none of the inflammatory hardness at the base so characteristic of the variolous pock; others had been torn away—probably by scratching—and showed shallow red pits. The temperature was 102 deg., there was constant vomiting, and intolerable itching of the skin.

I prescribed an effervescing mixture (potion Rivière) and 5 grs. of antipyrin every two or three hours, and an alkaline antiseptic lotion to subdue the excessive irritation of the skin.

Next morning the temperature was normal, but some of the papules on the face had become vesicular and turbid, and a fresh crop of papules and vesicles had appeared on the arms, and were threatening on the feet—as the patient complained of great heat and irritation, especially in the soles.

In the afternoon the fever was again 102 deg., and some more mixed eruption was now seen on the forearms as far as the wrists. The sickness was somewhat better, but there was occasional vomiting and no inclination for food, the tongue being rather furred, and the bowels constipated. Next day the eruption appeared on the scalp, the number of vesicles being considerable and causing much irritation. On the Monday (fifth day of eruption) the face and chest had cleared up, small shallow reddish pits showing up on the white skin, and some more mixed eruption had appeared on the insteps and lower parts of the legs and the burning sensation had disappeared from the soles of the feet. Temperature was normal all day, there was no more sickness nor lumbar pain, and the patient expressed herself as feeling quite well.

She got up on the seventh day, and a day or two later resumed her ordinary household duties. I forgot to say that on the Saturday (third day of eruption) the patient complained of some soreness in swallowing, but on examining the mouth I could only find some small hyperæmic patches chiefly on the soft palate.

There was constipation all through, relieved at first by enemata.

Remarks.—The interest of the case lies in: (1) The age of the patient; (2) distinct prodromal stage; (3) comparative violence of the eruptive stage; and (4) diagnosis.

1. Chicken-pox is rare in the adult. Fagge says that most writers have never seen it in grown-up people. I have seen one other case, strangely enough also in a young pregnant woman who happened to be in the obstetric ward at Guy's, under Dr. Galabin, some years ago (about 1878); the eruption in that case was also most marked on the chest, face, and arms.

2. The woman was unwell for some days preceding the eruption, and very ill on the day it came out. In children the prodromal stage is very slight, or passes unnoticed.

3. Coincident with the appearance of the rash came continuous and severe lumbar pain, and frequent vomiting and nausea, which lasted over three days.

The diagnosis was made a little difficult at first, owing to the history of four days of prodromal symptoms, and the appearance of the rash accompanied by lumbar pain and vomiting, all of which, in an adult especially, would point to small-pox. The successive crops were well marked, and the quickly changing papules and vesicles, the best guide: the temperature also remained high after the eruption had appeared, which is not the case in small-pox.

It will be interesting to see whether the child, when born, will show signs of having gone through the disease. The woman said she felt great "heat" in the uterus all through her illness.

